# Genome Sequence of vB_AbaS_TRS1, a Viable Prophage Isolated from *Acinetobacter baumannii* Strain A118

**DOI:** 10.1128/genomeA.01051-16

**Published:** 2016-10-13

**Authors:** Dann Turner, Matthew E. Wand, J. Mark Sutton, Daniela Centron, Andrew M. Kropinski, Darren M. Reynolds

**Affiliations:** a Centre for Research in Biosciences, Faculty of Health and Applied Sciences, University of the West of England, Bristol, United Kingdom; b National Infections Service, Public Health England, Salisbury, United Kingdom; c Instituto de Microbiología y Parasitología Médica (IMPaM, UBA-CONICET), Facultad de Medicina, Universidad de Buenos Aires, Buenos Aires, Argentina; d Departments of Food Science, Molecular and Cellular Biology, and Pathobiology, University of Guelph, Guelph, Ontario, Canada

## Abstract

A novel temperate phage, vB_AbaS_TRS1, was isolated from cultures of *Acinetobacter baumannii* strain A118 that had been exposed to mitomycin C. Phage TRS1 belongs to the *Siphoviridae* family of bacteriophages and encapsulates a 40,749-bp genome encoding 70 coding sequences and a single tRNA.

## GENOME ANNOUNCEMENT

Multidrug-resistant strains of *Acinetobacter baumannii* have emerged as prominent causative agents of nosocomial and community-acquired infections. To date, the complete genome sequences of 38 bacteriophages infecting the genus *Acinetobacter* have been deposited in the international nucleotide sequence databases comprising members of the *Myoviridae*, *Siphoviridae*, *Podoviridae*, and *Leviviridae* families. Many strains of *A. baumannii* are polylysogenic, harboring multiple integrated prophages (1), yet the influence of these upon host fitness and virulence is little understood.

*Acinetobacter baumannii* strain A118 was originally isolated from a blood culture in Buenos Aires, Argentina. Distinguishing features of this strain include a broad susceptibility to antibiotics and natural competence (2, 3). A draft genome sequence of A118 is available (whole-genome shotgun project AEOW01) (4). Phage TRS1 was induced from cultures of *A. baumannii* A118 by culturing in the presence of 1 µg·ml^−1^ mitomycin C and purified by CsCl density gradient centrifugation (5). Transmission electron microscopy revealed that TRS1 belongs to the family *Siphoviridae*. The virion particles possess an isometric head of 56-nm diameter and a noncontractile tail of 142-nm length that terminates in a tail tip and side tailspikes. Phage genomic DNA was sequenced using an Illumina HiSeq at the Genomic Services and Development Unit (Public Health England) with 100-bp paired-end reads and an average insert size of 338 bp. The genome was assembled using SPAdes version 3.5.0 (6) and resulted in a single circular contig of relatively homogeneous 1,419-fold coverage, indicating that the TRS1 genome is either circularly permuted or has direct terminal repeats. Open reading frames were predicted using a combination of GeneMark.hmm (7), Prodigal (8), and manual curation. Predicted functions for gene products were assigned using BLASTp (9), InterProScan (10), and HH-suite (11). Putative rho-independent terminators were predicted using TransTermHP (12) and assessed using MFold (13).

The TRS1 genome is 40,749 bp with a G+C content of 40.26% and encodes 70 proteins. A single tRNA for arginine with anticodon TCT was predicted using tRNAscan-SE version 2.09 (14). Of the 70 coding sequences, 36 were assigned a putative function and 34 were conserved hypothetical proteins. Like many other members of the family *Siphoviridae*, the TRS1 genome is organized into discrete modules comprising genes involved in DNA packaging, virion morphogenesis, lysis, integration, transcriptional regulation, and replication. The putative *attP* site was identified as a 29-bp sequence (TTATAAAATAGATTGGTGCGCTCGGCGGG) located upstream of a tyrosine family integrase. This sequence was also identified at the boundaries of putative prophages in the *A. baumannii* strains A1, SDF, LAC-4, and AB030. Alignment with the assembly data available for A118 revealed that the TRS1 genome sequence mapped across 11 contigs (GenBank no. AEOW01001344-55), and gap-fills between these contigs represented approximately 660 bp.

Genomic comparisons at the nucleotide level revealed little sequence identity between TRS1 and other phages. Greater nucleotide and protein similarity was observed among *Acinetobacter* spp. with sequenced genomes and consisted of limited regions of sequence similarity interspersed by nonhomologous regions, suggesting that TRS1 is highly mosaic.

### Accession number(s).

This whole-genome shotgun project has been deposited in GenBank under the accession number KX268652. The version described in this paper is the first version, KX268652.1.
